# Simulating the Commercial Implementation of Gene-Editing for Influenza A Virus Resistance in Pigs: An Economic and Genetic Analysis

**DOI:** 10.3390/genes13081436

**Published:** 2022-08-12

**Authors:** Hamish A. Salvesen, Timothy J. Byrne, C. Bruce A. Whitelaw, Fiona S. Hely

**Affiliations:** 1 The Roslin Institute and Royal (Dick) School of Veterinary Studies, University of Edinburgh, Easter Bush EH25 9RG, UK; 2 AbacusBio International Limited, The Roslin Innovation Centre, Edinburgh EH25 9RG, UK; 3 AbacusBio Limited, 442 Moray Place, Dunedin 9016, New Zealand

**Keywords:** gene-editing, influenza A virus, CRISPR, mosaicism

## Abstract

The development of swine Influenza A Virus resistance along with genetic technologies could complement current control measures to help to improve animal welfare standards and the economic efficiency of pig production. We have created a simulation model to assess the genetic and economic implications of various gene-editing methods that could be implemented in a commercial, multi-tiered swine breeding system. Our results demonstrate the length of the gene-editing program was negatively associated with genetic progress in commercial pigs and that the time required to reach fixation of resistance alleles was reduced if the efficiency of gene-editing is greater. The simulations included the resistance conferred in a digenic model, the inclusion of genetic mosaicism in progeny, and the effects of selection accuracy. In all scenarios, the level of mosaicism had a greater effect on the time required to reach resistance allele fixation and the genetic progress of the herd than gene-editing efficiency and zygote survival. The economic analysis highlights that selection accuracy will not affect the duration of gene-editing and the investment required compared to the effects of gene-editing-associated mosaicism and the swine Influenza A Virus control strategy on farms. These modelling results provide novel insights into the economic and genetic implications of targeting two genes in a commercial pig gene-editing program and the effects of selection accuracy and mosaicism.

## 1. Introduction

Influenza A virus (IAV) is a significant pathogen of humans and several keystone agricultural species, such as chickens and pigs. Its global distribution and ability to cross zoonotic barriers contribute to its potential as a source for emergent pandemics [1]. This pandemic potential is exemplified by the swine-originating 1918 Spanish ‘Flu pandemic that is estimated to have claimed 50–100 million lives [2]. Having effective control measures to reduce IAV prevalence and transmission in swine herds will assist in mitigating the emergence of another pandemic strain [3]. Furthermore, although annual epidemics of swine IAV (swIAV) have low mortality rates, high morbidity rates are associated with lower animal welfare standards and reduced productivity that ultimately affects economic performance of the pig industry [4,5]. With a global herd-level seroprevalence of 72.8%, swIAV is an endemic problem faced by most hog farmers [6]. The industrial expansion of pig farming has been associated with an increased swIAV prevalence [6], and a continuation of this trend will therefore likely contribute to an increasing prevalence.

With increasing swIAV prevalence, the likelihood of two distinct strains infecting a single host grows. In the event that multiple strains of IAV co-infect a host, the eight, segmented RNA genomes of IAV can be reassorted [7,8]. Genomic reassortment generates a novel virus subtype, one that may have improved potential for intraspecies or zoonotic transmission into naïve hosts [9,10]. The difficulty of controlling swIAV stems from its heterogeneity and ability to rapidly evolve. Removing pigs as a reservoir for IAV infection will have the dual benefit of reducing the burden of disease in pigs and reducing the potential for pandemic emergence through genomic reassortment.

Because swIAV has a low mortality rate, there is a large amount of variability in the application of control measures [11]. Herd management and basic biosecurity are the most widely applied measures, with quarantine of new arrivals and cleansing of pens between stock movements amongst the simplest methods. Where industrialised piggeries have been adopted, there is a wider uptake of proactive control in the form of vaccination programs [12]. Success of vaccination programs is variable due to the intrinsic evolutionary capability of swIAV. Additionally, because only endemic swIAV strains are targeted, vaccination does not prevent human-swine transmission [13]. With a limited arsenal of swIAV control techniques available, it is important we critically appraise the tools at our disposal. Genetic-based technologies such as gene-editing offer a novel and proactive control strategy that would complement current measures [14].

As an intracellular parasite, IAV relies on host proteins to support their limited complement of proteins and therefore to complete their life cycle [15,16]. Its reliance on host factors means that disruption of virus–host protein interactions by alteration of specific amino acids could impede viral replication, thereby reducing infection and/or transmission. Targeted and specific changes to the DNA sequence can be made using gene-editing technologies such as CRIPSR/Cas9 [17]. Examples of CRISPR/Cas9 being utilised against viral infections in livestock includes pigs resistant to Porcine Reproductive and Respiratory Syndrome virus (PRRSv) and Transmissible Gastroenteritis virus (TGEV), as well as resistance in chickens to avian leukosis virus [18,19,20]. Identified genotypes which confer resistance to viral pathogens in pigs are haploinsufficient, and therefore successful editing of both alleles is necessary for full resistance [18,19]. In vitro data from human and avian cell models suggests that by application of the same principles to IAV-relevant genes, there is promise for the creation of swIAV-resistant pigs [21,22].

Modelling the economic repercussions, including the opportunity cost of less genetic improvement from selecting for viral resistance alleles and the direct costs of a gene-editing program against the benefits of improved productivity from swIAV resistance and reduced veterinary costs from the generation and use of swIAV-resistant pigs in commercial pig production, is an important step in understanding the value proposition of gene-editing in commercial pigs. We have modelled the introgression of swIAV resistance alleles in a multi-tiered pig population, whereby editing a single gene confers full resistance (monogenic), as observed with PRRSv, and where digenic gene-editing on either the same or discrete chromosomes is required for full viral resistance.

**Figure 1:**
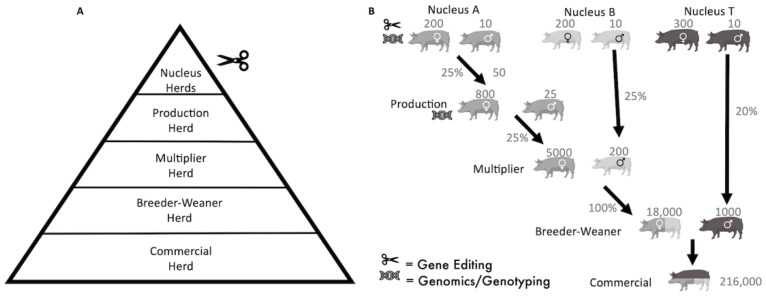
Outline of commercial pig breeding systems as designed in the simulations. (**A**) Schematic representation of the pyramidal structure and the herds and tiers of a commercial pig breeding system as simulated, styled on a pyramidal breeding system as described in Visscher et al., 2000. (**B**) Breeding population structure and dynamics used in our simulation model. Numbers above the pigs indicate the number of boars/dams used for breeding in each batch. Percentages indicate the proportion of available females from the tier above that are transferred down a tier.

From the available literature, we have not identified a model for integrating alleles by gene-editing into a multi-tiered pig breeding pyramid, and for other species a digenic model has not been published [23,24]. In the pyramid breeding structure employed in commercial pig breeding, gene-editing could occur only in the top breeding tier, with alleles flowing down by selection to the Finisher herd at the base (Figure 1A), making it a particularly efficient breeding system for allele dissemination.

**Figure 2:**
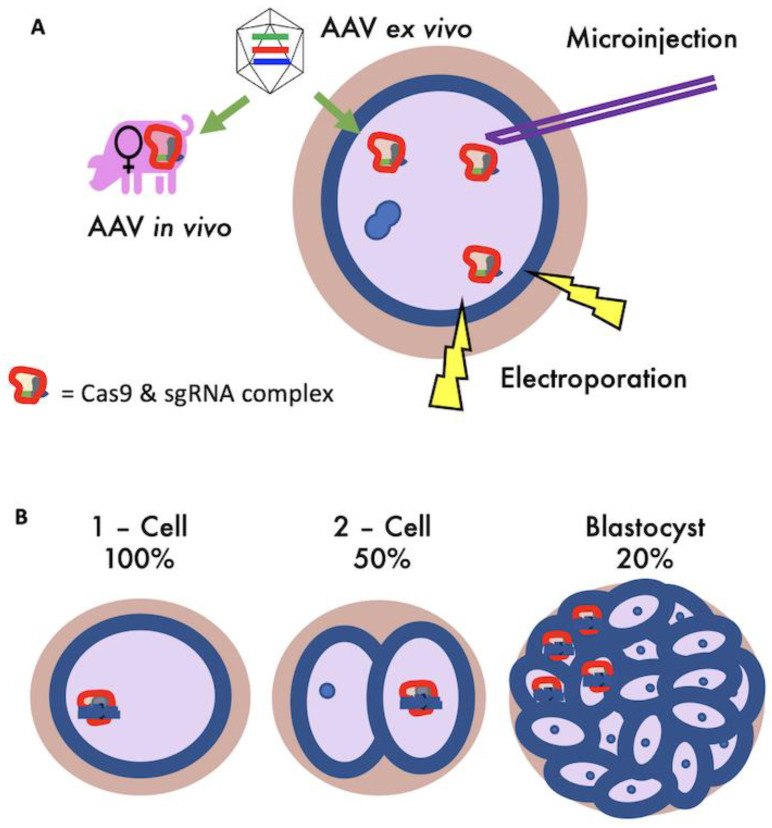
Schematic representations of gene-editing techniques considered for commercial applications and gene-editing introduced mosaicism. (**A**) Gene-editing methods applied to porcine zygotes. Green arrows indicate the addition of Adeno-associated Virus (AAV) vectors either to embryos in vitro or directly in utero. (**B**) The stochastic distribution of gene-editing reagents during embryonic division or delayed and asymmetrical CRISPR/Cas9 activity can lead to a reduced likelihood of germline transmission as a result of mosaicism. Gene-editing at a one-cell stage will result in non-mosaic progeny. Gene-editing in a single cell after a single cell division will result in a 50% mosaic organism, and gene-editing at a later embryonic stage will result in the relevant proportion of a progeny’s cells having the desired genetic alterations, which may include the germline progenitor cells.

Our simulation model considered four methods of getting CRISPR/Cas9 gene-editing reagents into zygotes (Figure 2A) [25]; (1) microinjection [26], (2) electroporation [27], and transduction of zygotes with recombinant adeno-associated virus (AAV) vectors, performed on zygotes (3) ex vivo or (4) in vivo [28,29]. These methods have different efficiencies of gene-editing, rates of zygote death, and procedural costs. All simulation parameters are based on CRISPR/Cas9 data for gene-editing by Non-Homolgous End Joining (NHEJ), using one single guide RNA (sgRNA) for each target gene.

Microinjection is well-established in pigs as a method of introducing gene-editing reagents into zygotes by physically injecting the reagents by needle penetration [26]. Electroporation works by transiently disrupting the zona pellucida and zygote membrane with electrical impulses, allowing the movement of gene-editing reagents from the surrounding solution [27]. Electroporation is less well-established in a research setting, but is more commercially attractive due to its capacity for high-throughput and generally higher gene-editing efficiency. Transduction of zygotes with AAV vectors, performed on zygotes ex vivo or in vivo, has to date only been performed in rodent species [28,29]. If AAV reagents can be optimised for use on pig zygotes, the relatively low skill and cost requirements alongside its capacity to be scaled up could make it particularly appealing commercially [25]. Furthermore, in vivo AAV could be implemented alongside artificial insemination (AI) procedures, making it a seamless procedural change for current breeding programs. Given that experimental results for gene-editing methods in zygotes are highly variable, the values identified from the literature and assigned as parameters in this simulation model are illustrative.

The relatively low skill and cost requirements of AAV, alongside its capacity to be scaled up, could make it particularly appealing commercially [25]. Furthermore, in vivo AAV could be implemented alongside artificial insemination (AI) procedures, making it a seamless procedural change for current breeding programs. Given that experimental results for gene-editing methods in zygotes are highly variable, the values identified from the literature and assigned as parameters in this simulation model are illustrative.

An important factor not included in previous livestock gene-editing simulation models is genetic mosaicism [30,31,32]. Mosaicism occurs during embryogenesis when a mutation happens after the first cell division, leading to cellular descendants having different genotypes to their ancestors [18,33] (Figure 2B). The phenomenon of mosaicism impacts the heritability of gene-editing because transmission of the novel allele is disrupted if the changes made to DNA are not present in the germline stem cells. Here, mosaicism is referred to specifically in the context describing the level of germline transmission.

The simulation models recorded the level of gene-editing required to reach genotypic and phenotypic fixation in the Finisher herd of a commercial pig breeding system. To compare prevailing gene-editing methods, we assessed varying gene-editing efficiencies and zygote death rates under different levels of mosaicism. A comparative economic analysis was carried out to assess the trade-offs and the financial capacity required to deploy a gene-editing program in a commercial pig breeding system.

The findings of these simulation models highlight some of the economic and genetic considerations for the implementation of gene-editing in commercial pig herds. Reducing the amount of genetic mosaicism associated with the gene-editing process for the target genes will offer the largest improvements in outcomes associated with gene-editing programs in a multi-tiered pig herd. The economic analysis suggests that the presence of a vaccination program will be a major determinant of whether breeding programs will be financially incentivised to incorporate gene-editing for swine Influenza A Virus resistance.

## 2. Materials and Methods

This simulation model was designed to assess the flow of gene-edited alleles through a multi-tiered commercial pig breeding pyramid based upon a three-breed and five-tiered pyramid breeding structure (Figure 1) [34,35]. Selected methods of gene-editing were assessed with variable levels of mosaicism. The model was developed using R software (R Core Team, Vienna, Austria). The code is available in the GitHub repository (https://github.com/hamishsalvy/SwineFluGene-Editing, accessed on 8 August 2022). All data visualisations were created using the plotly package (R Studio) with the mean values taken from 10 iterations for each gene-editing method with independent mosaicism levels and selection accuracies.

### 2.1. Base Population

Initially, a population of Nucleus pigs without swIAV resistance alleles was created and split into 3 breeds, “A”, “B” and “T” (Figure 1B). Simulations were performed, assuming herd management in batches. Each batch was defined as 28 days, which allowed for the assumption of 4 batches (112 days) to be a dam pregnancy length and 1 batch to be the lactation period of piglets and the return to oestrus period [36]. These periods will vary slightly by breed, farm and management, but consistent modelling meant dams could be selected for breeding every 5 batches and remained representative of breeding swine cycles [34]. Each batch was distinct, with mating only occurring on day one. Breeding age boars and gilts (>8 batches old [36]) were made available for selection every batch and culled after 38 and 42 batches, respectively. Random mortality of all pigs over 1 month of age was applied at 2.5% every batch. A summary of the breeding parameters used are presented in Table 1.

Mating pairs were selected according to their genetic merit, determined in a nested design by sorting eligible boars and females in descending order of their genetic merit value. For example, in the “A” Nucleus population, 200 females were selected for mating in each generation. The 10 top boars were crossed with the top 10 females, with each sex ordered by descending genetic merit. Each subsequent group of 10 ordered females was bred with the initial 10 boars. This is known as a nested breeding design [37]. The “T” Nucleus population supported 300 females to ensure enough boars are available for natural breeding with the Breeder-Weaner tier. The selection parameters for breeding animals and the numbers/proportion of pigs moving down the pyramid are described in Figure 1B.

Piglets had an equal probability for sex assignment and alleles were inherited according to Mendelian principles. Founder pigs created for the Base Population pigs were assigned a Breeding Value (BV) by drawing a random variate from a normal distribution with a mean of 0 and standard deviation of 10 [38]. This breeding value was assigned as an aggregated ‘genetic merit’ and not by specific trait indexing. Each piglet was assigned a BV from half of the combined maternal and paternal value, plus a Mendelian sampling term. Selection was based on a genomic prediction of these BVs, where the genomic prediction had a heritability of 1 [39] and the accuracy of the genomic prediction was set at 1, 0.8 or 0.5 by scaling the genetic standard deviation (indexSD-10) used in the BV estimation by the genomic prediction accuracy.

To establish the pyramidal structure, breeding within the Nucleus tier was simulated for 20 batches before the Production tier was initiated. After 45 batches, flow down to the Multiplier tier began, followed by the Breeder-Weaner tier after 55 batches. After 100 batches the pyramidal structured base population used for all forward simulations was established. Piglets were born into their parental tier and could only be present in a single tier. Mating of pigs in the Nucleus and Production tiers were simulated as artificial insemination (AI), with boars used concurrently in these tiers, whilst the Multiplier and Breeder-Weaner tiers were mated by conventional breeding, meaning boars could only be available for selection in a single tier for each batch.

### 2.2. Forward Simulations

Using the established base population, four gene-editing methods were applied to confer monogenic or digenic resistance to swIAV. For full resistance to viral infections, both alleles were required to be present. The inheritance mode of digenic resistance was either linked (with no meiotic recombination) or unlinked to inheritance of resistance genes. Each simulation ran for 120 batches (~10 years).

Selection in the Nucleus and Production tiers was based on a point being assigned to each allele, creating an individual genotype score for each pig. Wildtype animals equaled 0 and digenic resistant animals equaled 4. The designated percentage or number of breeding animals were primarily selected according to their allele score, followed by selecting the top fraction of eligible mating boars and sows by ranking on genetic merit. Resistance alleles were only selected for in the Nucleus and Production tiers, where genotyping is carried out. In the Multiplier and Breeder-Weaner tiers only the genetic merit values from pedigree geneflow were considered to determine breeding females. The Finisher herd was included for forward simulations.

### 2.3. Gene-Editing & Mosaicism

Gene-editing was applied to zygotes with wildtype alleles in the Nucleus A, B and T populations. The relevant parameters for each gene-editing method are outlined in Table 2. The estimated costs of gene-editing include the pricing of reagents, embryo transfer, labour and animal husbandry to the point of piglet birth. For AAV-based techniques, murine data was used as gene-editing efficiencies and zygote survival data was unavailable for porcine zygotes.

Gene-editing was performed to all zygotes from mating pairs with at least one swIAV susceptibility allele, with the editing efficiency applied to zygote alleles individually and the death rate applied to zygotes post-editing and implantation. Mosaicism was included by reducing the proportion of successfully gene-edited alleles that are present in each animals germline (20%, 50% or 100%). By example, for 20% mosaicism, 20% of progeny will have correctly gene-edited alleles in their germline (Figure 2B).

### 2.4. Economic Analysis

The economic analysis was built on selected cost and benefit components associated with implementing gene-editing to generate swIAV-resistant pigs. This included the direct costs of gene-editing (such as having less pigs reaching slaughter due to zygote deaths) and a reduction in genetic progress (i.e., growth efficiency, maternal traits and carcass traits) arising from diverted selection pressure, against the financial benefit derived from improved productivity and reduced veterinary costs. The parameters used in the economic analysis are described in Table 3, with all $ values designated in United States Dollars (USD).

The annual cost of editing was determined by multiplying the number of attempted zygote gene-edits by the cost of gene-editing per zygote. Costs of gene-editing were extrapolated from research lab data on gene-editing of porcine zygotes (personal communication, Chris Proudfoot). Each zygote death was a pig that could no longer be reared for slaughter and was therefore counted as lost revenue. The price of a finished pig was determined as $109.5, a ten-year mean of whole hog value in the USA (2010–2019) [40]. The cost of swIAV in pigs, accounting for the co-morbidities of Porcine Respiratory Disease Complex (PRDC), has been estimated to be $10.31 [41]. The reduction in the genetic merit of the Finisher herd from biased selection towards swIAV resistance alleles was determined as a monetary value using

Lost Merit ($)=Z∗Base(t)∗number of commercial pigs slaughtered 

(*Z* = proportion of genetic gain compared to control, *Base* = Annual genetic improvement in profit per pig, *t* = year). It was assumed that the potential for an annual genetic gain of $4 remained consistent over the entire simulation period.

The financial benefit derived from having swIAV-resistant pigs was termed health benefit. For farms with vaccination, prior to gene-editing these farms still achieve an IAV-free productivity boost through the vaccination program. Here, the health benefit is the difference between the productivity boost and vaccination cost, which is applied only after the threshold of Herd Immunity (HI) is reached and vaccination can be stopped. For systems without vaccination, improved productivity was added for all phenotypically swIAV-resistant pigs, and subsequently to all pigs after the HI threshold was reached. HI was calculated as 90% using HI = (R0 − 1)/R0 [42]. R0 of swIAV transmission in unvaccinated pigs calculated to be 10.66 [43].

Annual costs were summed to generate a Real Value. The Real Value was multiplied by a discount factor (based on inflation of 5% (*r*)) to account for the financial opportunity cost and interest payments to determine a Present Value for each year (*t*) [44]. The present value was captured over the ten years to produce a cumulative Net Present Value (*NPV*), as:

$$NPV=\overset{n}{\underset{t=1}{\sum }}\;\times \;\frac{1}{{\left(1+r\right)}^{t}}$$

## 3. Results

The results presented illustrate how different gene-editing parameters and gene-editing-associated mosaicism will affect the flow of gene-edited alleles and genetic progression in a multi-tiered pig breeding pyramid. Further to the genetic facet of these simulations, our economic analysis outlines the considerations breeders should consider when determining whether it is effective to implement a gene-editing program for swIAV resistance.

**Figure 3:**
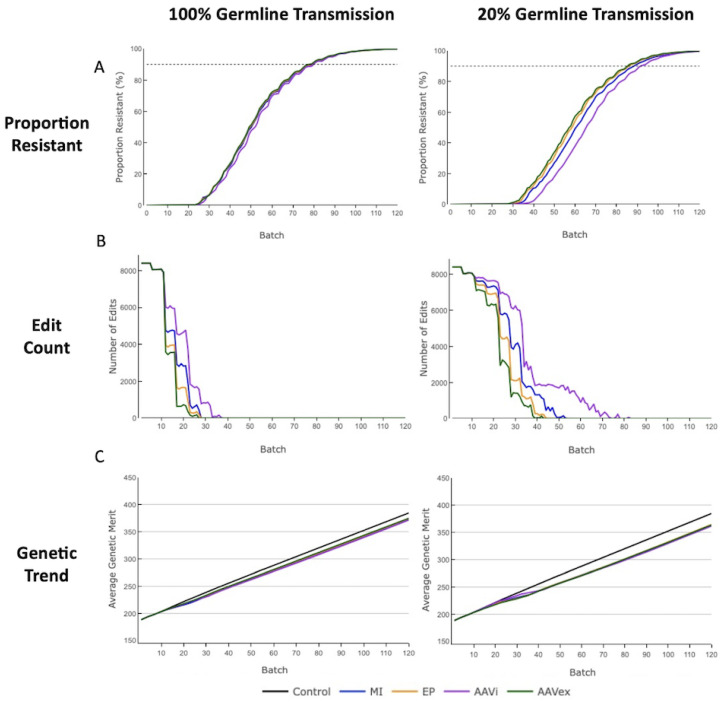
Monogenic swIAV resistance with 100% or 20% germline transmission with a selection accuracy of 0.8. MI = Microinjection. EP = Electroporation. AAVi = AAV in vivo. AAVex = AAV ex vivo. (**A**) Proportion of pigs with phenotypic resistance to swIAV in the Finisher herd. The dashed horizontal line at 90% represents the herd immunity threshold. (**B**) The number of zygotes that were attempted to be gene-edited in all Nucleus tiers per batch. (**C**) The mean genetic merit of pigs in the Finisher herd.

When targeting a single gene, the proportion of phenotypically swIAV-resistant pigs in the Finisher herd reached the HI threshold (90%) within 120 batches for all gene-editing methods at differing levels of mosaicism and had a delay associated with 20% mosaicism compared to 100% transmission (Figure 3). For 50% mosaicism the delay was intermediary (Supplementary Figure S1). Monogenic data displayed is for simulations applying a the moderate-high selection accuracy of 0.8. Only the trend of genetic merit, and not the dissemination of alleles through the tiers of the breeding pyramid or the amount of gene-editing required was affected when adjusting selection accuracy (Supplementary Figure S2).

The proportion of swIAV-resistant pigs in the Finisher herd aligned by decreasing efficiency of gene-editing; AAV ex vivo, electroporation, microinjection, AAV in vivo. For 100% mosaicism there were only small differences in time to reach HI between each gene-editing method (<2%), with outcomes becoming more divergent with 20% mosaicism (<6%) (Figure 3A). AAV in vivo had the largest increase in the time taken to reach HI when changing from 100% to 20% mosaicism, with an increase to the mean of 11 batches (14%), whereas the mean number of batches for AAV ex vivo increased by 6 (8%).

The attempted zygote gene-edits also aligned according to decreasing gene-editing efficiency (Figure 3B). For lower efficiency gene-editing methods, increasing mosaicism, and thereby reducing the germline transmission of gene-edited alleles had a more pronounced impact on the volume of gene-editing required. Moving from 100% to 20% mosaicism there was an increase to the mean volume of zygotes gene-edited of 68% for AAV ex vivo, 74% for electroporation, 80% for microinjection and 89% for AAV in vivo. For AAV in vivo there was an increase of 44 to the mean number of batches that gene-editing was performed for between 100% and 20% mosaicism, whereas the mean number of batches that gene-editing was performed for was increased by 16 with the more efficient AAV ex vivo method.

For all gene-editing methods there was a greater reduction in genetic progress after 120 batches with 20% mosaicism than for 100% mosaicism when compared to the control population (Figure 3C). With 100% mosaicism there was a 2.5–3.1% reduction in the mean genetic merit value across all gene-editing methods, compared to the control population after 120 batches and for 20% mosaicism where there was a 5.2–6% reduction. With a selection accuracy of 0.5, the reduction in mean genetic merit across the gene-editing methods is 2.1–3% for 100% mosaicism and 4–4.9% for 20% mosaicism, illustrating that a smaller reduction to genetic improvement was observed with lower selection accuracies (Supplementary Figure S3).

### 3.1. Digenic Modelling

The digenic model in this simulation required four resistance alleles to be present for phenotypic resistance and no viral escape mutants were included in the simulation or analyses.

### 3.2. Proportion Resistant

**Figure 4:**
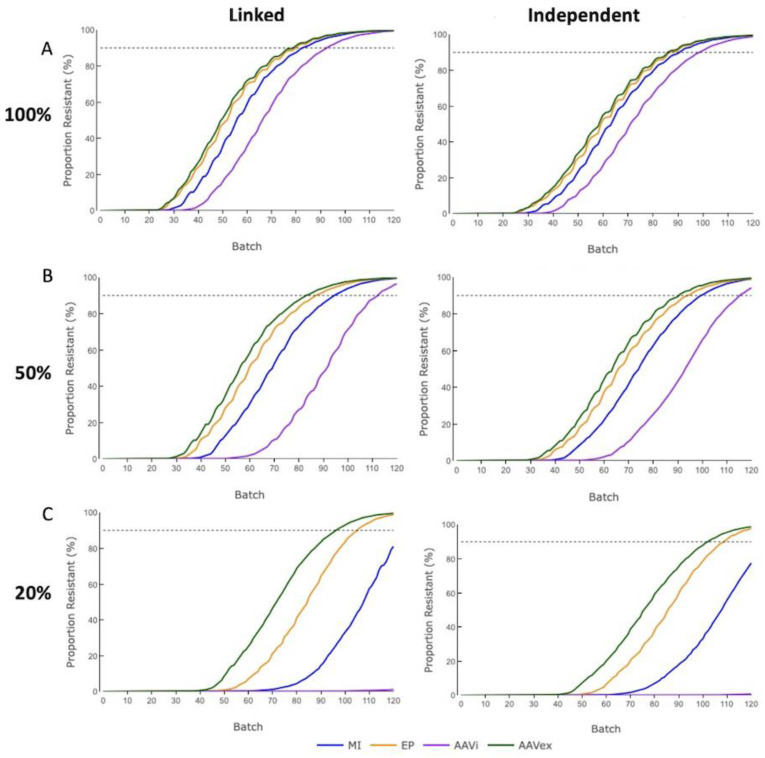
The proportion of swIAV-resistant pigs in the Finisher herd in a digenic gene-editing program with a selection accuracy of 0.8. MI = Microinjection. EP = Electroporation. AAVi = AAV in vivo. AAVex = AAV ex vivo. Influenza resistance alleles were inherited in a completely linked or independent manner. (**A**) 100% germline transmission. (**B**) 50% germline transmission. (**C**) 20% germline transmission.

The proportion of resistant animals in the Finisher herd was counted at the end of each batch to observe the time over which resistant animals filtered down to the commercial growers (Figure 4). The dissemination of resistance alleles down the breeding pyramid was not affected by changing selection accuracy between 1, 0.8 and 0.5 (Supplementary Figure S3).

For all gene-editing methods, the accumulation of phenotypically resistant pigs was delayed when resistance alleles were inherited independently compared to when resistance alleles were in complete linkage. With 100% or 50% mosaicism, Finisher herds reached the threshold for HI of 90% within the 120 batches under all gene-editing methods. With 20% mosaicism, only the more efficient AAV ex vivo and electroporation techniques reached the HI threshold for both digenic inheritance modes within 120 batches, and swIAV-resistant pigs from the lowest efficiency AAV in vivo cohort were only just beginning to appear in the Finisher herd. With 100% mosaicism, the most efficient gene-editing method of AAV ex vivo reached the HI threshold 7 batches (10%) later in instances where resistance alleles were independently inherited versus those where they were in complete linkage. Conversely, for the least efficient method of AAV in vivo, there was a smaller increase of 6 batches (6.5%).

For AAV in vivo, the resistance phenotype was just beginning to emerge in the Finisher herd after 120 batches with 20% mosaicism whilst microinjection would reach HI just beyond simulated timeframe. These results suggest that implementing gene-editing with parameters, similar to the AAV in vivo values used in these models, would make it an unfeasible method in a commercial pig breeding system if mosaicism levels were as low as 20%.

### 3.3. Edit Count

**Figure 5:**
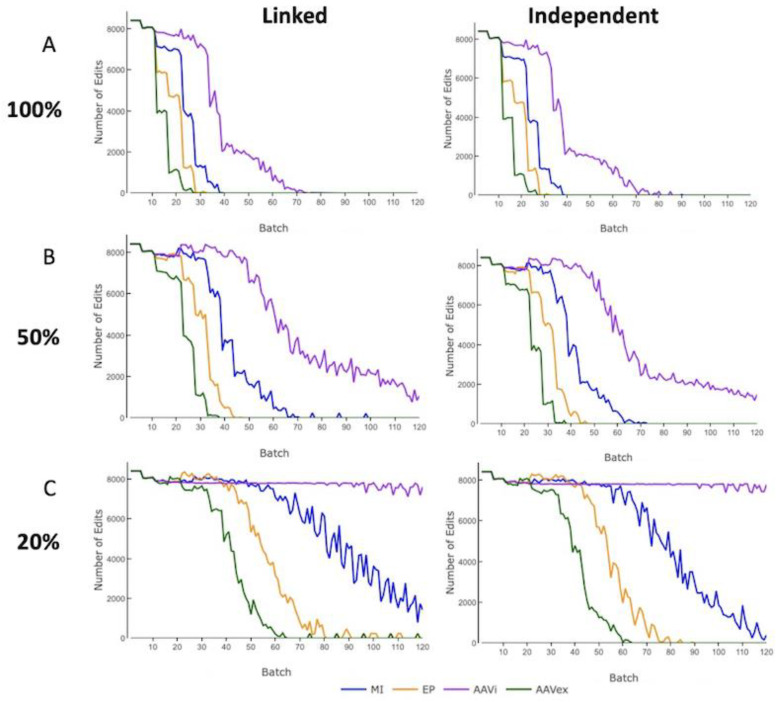
The proportion of swIAV-resistant pigs in the Finisher herd in a digenic gene-editing program with a selection accuracy of 0.8. MI = Microinjection. EP = Electroporation. AAVi = AAV in vivo. AAVex = AAV ex vivo. Influenza resistance alleles were inherited in a completely linked or independent manner. (**A**) 100% germline transmission. (**B**) 50% germline transmission. (**C**) 20% germline transmission.

The count of zygotes that were gene-edited across all Nucleus populations was recorded per batch. No gene-editing occurred when only swIAV resistance alleles were present in the Nucleus Herd animals that were selected for breeding. For both linked and independent inheritance across all levels of mosaicism, the number of zygotes gene-edited aligns in order of descending gene-editing efficiency for a selection accuracy of 0.8 (Figure 5). There was no observable effect to the level of gene-editing required when changing the level of selection accuracy (Supplementary Figure S4).

At 100% mosaicism, for AAV in vivo the mean number of zygotes that were attempted to be gene-edited across the 120 batches was 2.7% higher for independently inherited alleles than linked alleles, with all other gene-editing methods having <0.2% discrepancy between inheritance modes. Selected Nucleus breeding animals were fixed for swIAV resistance alleles within 27 batches for AAV ex vivo, 32 for electroporation and 41 for microinjection at 100% mosaicism for linked or independent inherited alleles. For AAV in vivo, there was a long tail of persistent gene-editing and the Nucleus breeding animals did not reach fixation for swIAV resistance alleles until 87 batches.

With 20% mosaicism, only AAV ex vivo and electroporation reached the resistance allele fixation within 120 batches and there was <3% difference in the mean number of zygotes gene-edited over 120 batches between linked or independently inherited alleles. For AAV ex vivo and electroporation, moving from 100% to 50% mosaicism resulted in an increase of 61% and 63%, respectively, for both linked and independently inherited alleles. Changing mosaicism from 50% to 20% mosaicism resulted in the mean number of zygotes being gene-edited, increasing by 74% for AAV ex vivo with linked alleles and 80% for independently inherited alleles. These results highlight the challenges presented by high levels of mosaicism as a result of the increased amount of gene-editing required from mosaicism.

### 3.4. Genetic Merit Trend

**Figure 6:**
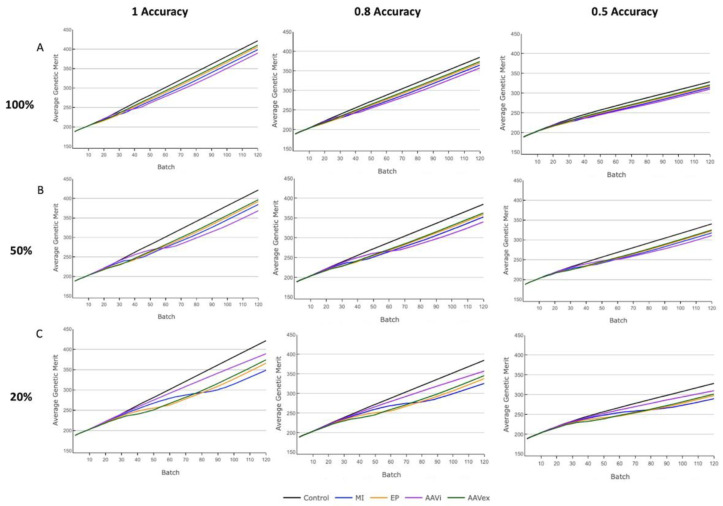
Genetic merit trend of piglets in the Finisher herd in a digenic gene-editing program with varying selection accuracies. MI = Microinjection. EP = Electroporation. AAVi = AAV in vivo. AAVex = AAV ex vivo. Influenza resistance alleles were inherited in an independent manner. (**A**) 100% germline transmission. (**B**) 50% germline transmission. (**C**) 20% germline transmission.

The trend in genetic merit in the Finisher herd was measured to assess the impact of prioritising the selection of resistance alleles over an index of genetic merit for the Nucleus and Production tiers (Figure 6). The mode of inheritance did not affect the genetic merit index value after 120 batches, as observed by alleles inherited in complete linkage being within 2 index points of independently inherited alleles after 120 batches for 100% and 50% mosaicism, and 5 points for 20% mosaicism (Supplementary Figure S5). For all selection accuracies, the mean genetic merit after 120 batches was reduced as compared to the unedited control population in alignment with decreasing gene-editing efficiency (except for AAV in vivo at 20% mosaicism).

This result was hypothesised because, when resistance alleles are more prevalent in breeding animals, selection can be more focused on genetic merit index values. The AAV in vivo exception with 20% mosaicism occurs because so few swIAV resistance alleles are present in breeding animals after 120 batches. Therefore, the rate of improvement in index genetic merit will continue to reduce beyond the endpoint of these simulations as bias towards swIAV resistance allele selection increases in accordance with their allele frequency. As selection accuracy was decreased the difference in index genetic merit values between each gene-editing method after 120 batches was reduced (Figure 6).

Across all selection accuracies, the reduction in genetic merit after 120 batches increased when compared to the control population as the level of gene-edited alleles transmitting to the germline decreased due to mosaicism increasing. For example, under a selection accuracy of 1, AAV ex vivo had a 2.6% reduction in mean genetic merit with 100% mosaicism, 5.9% for 50% mosaicism and 11.2% with 20% mosaicism, whilst microinjection had a 5.2%, 8.6% and 17% reduction for 100%, 50% and 20% mosaicism, respectively. Electroporation reported values intermediate to those of AAV ex vivo and microinjection for all selection accuracies and mosaicism rates. AAV in vivo was an exception to this pattern, with 20% mosaicism above 50% mosaicism due to the low level of swIAV resistance alleles created throughout the 120 batches simulated.

### 3.5. Economic Analysis

**Figure 7:**
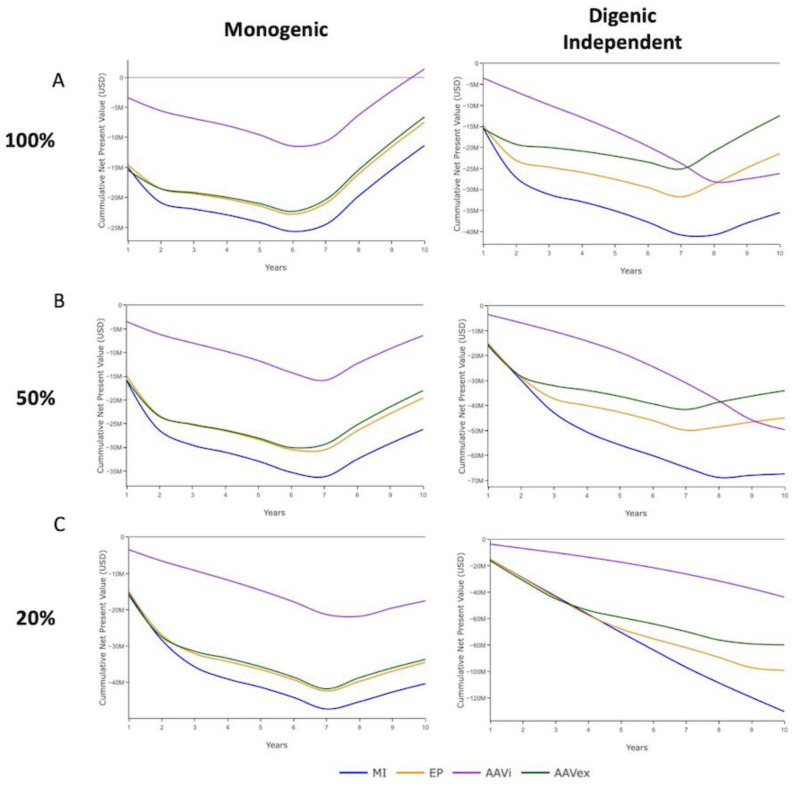
Economic analysis of farm systems with vaccination programs for monogenic and independently inherited digenic swIAV resistance alleles with a selection accuracy of 0.8. MI = Microinjection. EP = Electroporation. AAVi = AAV in vivo. AAVex = AAV ex vivo. The cumulative financial benefits of resistance outweigh the cumulative costs in USD of implementation once the line is above 0. (**A**) 100% germline transmission. (**B**) 50% germline transmission. (**C**) 20% germline transmission.

**Figure 8:**
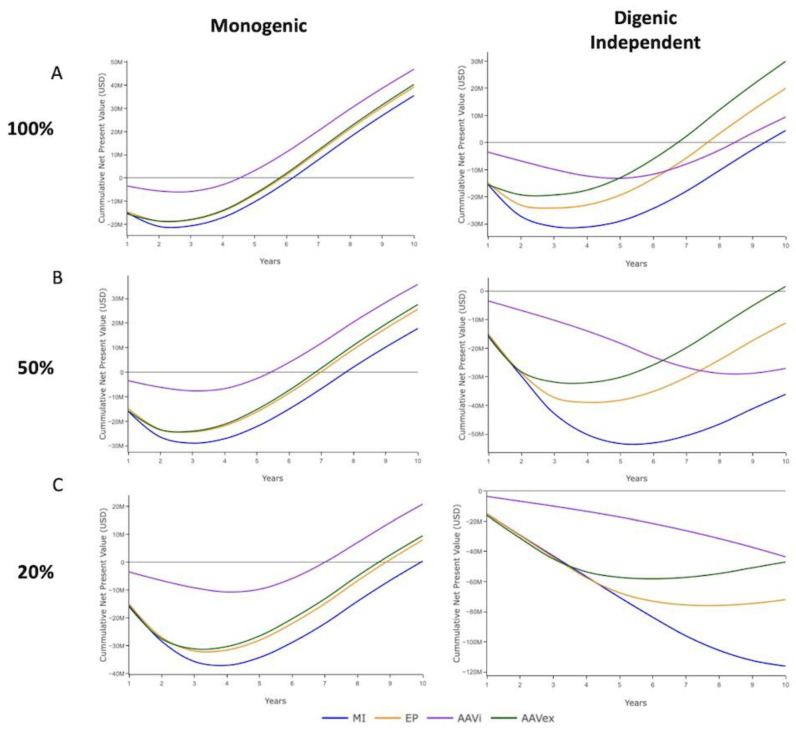
Economic analysis of farm systems with no vaccination program present for monogenic and independently inherited digenic swIAV resistance alleles with 0.8 selection accuracy. MI = Microinjection. EP = Electroporation. AAVi = AAV in vivo. AAVex = AAV ex vivo. The cumulative financial benefits of resistance outweigh the cumulative costs in USD of implementation once the line is above 0. (**A**) 100% germline transmission. (**B**) 50% germline transmission. (**C**) 20% germline transmission.

The economic analysis was designed to illustrate how the biological process of gene-editing and economic factors intertwine to influence decision making and the value proposition surrounding the implementation of a commercial gene-editing program. Decisions regarding the utilisation of gene-edited pigs will be affected by the swIAV control measures in place, so the analysis was split into systems with vaccination programs (Figure 7) that assumed ubiquitous and effective vaccination, and those with minimal swIAV control measures in place (Figure 8). The output for a selection accuracy of 0.8 and independent inheritance of digenic target alleles is shown to represent a moderate-high selection index accuracy in a discrete digenic model. Adjusting selection accuracy did not have a large effect on the economic analysis with the parameters used for these simulations (Supplementary Figures S6 and S7).

With vaccination, the economic benefits accrue when 90% of pigs are swIAV-resistant and vaccination is no longer required. Farm systems without vaccination benefit prior to this from improved productivity in individually swIAV-resistant pigs, and subsequently through productivity improvements to the entire herd once HI is achieved [45].

For production systems with robust vaccination schemes, only a monogenic target with gene-editing by AAV in vivo at 100% mosaicism achieved a positive cumulative NPV within 120 batches (Figure 7A). In no other scenarios was a positive cumulative NPV reached. As the number of gene-edited alleles present in the germline of progeny decreased due to the increased presence of mosaicism, the cumulative costs from extended gene-editing programs increased the projected time to reach a return on the initial capital investment under all scenarios. When gene-editing digenic targets, AAV ex vivo with 100% mosaicism had the smallest negative cumulative NPV and was projected to reach positivity soonest (Figure 7A). The introduction of a second swIAV resistance gene to the gene-editing scheme necessitated a much greater capital investment for all gene-editing methods and levels of mosaicism.

In farming systems that were simulated to have endemic swIAV and not have implement effective control measures, in the instance of monogenic resistance, all methods except microinjection with 20% mosaicism reached a positive cumulative NPV within the 10 years simulated (Figure 8). In order of time required to reach a positive cumulative NPV, AAV in vivo was the fastest, followed by AAV ex vivo and electroporation with similar projections, and finally microinjection. With 100% mosaicism, AAV in vivo, AAV ex vivo and electroporation reach a positive cumulative NPV within 6 years, which increased to 7 years for AAV in vivo and 9 years for AAV ex vivo and electroporation with 20% mosaicism.

For the digenic models in farm systems with endemic swIAV and no effective control measures, at 100% mosaicism all methods of gene-editing reached a positive cumulative NPV within the 10 years simulated. AAV ex vivo was the most cost effective, followed by electroporation, AAV in vivo and microinjection. With mosaicism of 50%, only AAV ex vivo reached a positive cumulative NPV within the 10 years simulated. For 20% mosaicism, negative cumulative NPVs were reported over the 10 years for all gene-editing methods simulated, with only AAV ex vivo and electroporation beginning to trend towards a positive value. These economic analyses outline some of the considerations in terms of biological optimisation of gene-editing protocols that should be taken into account when looking to integrate gene-editing into commercial pig breeding system.

## 4. Discussion

The simulation models presented here provide a novel analysis of the genetic and economic considerations when implementing a gene-editing program in a commercial pig breeding. system. The inclusion of digenic resistance and mosaicism provides further insight into the flow of resistance alleles that adhere to the biological reality of gene-editing in mammalian livestock for viral resistance that has not previously been published.

### 4.1. Monogenic Modelling

In the genetic analysis of the monogenic modelling there are only small changes in the time to reach fixation and in the progression of genetic merit between the methods of gene-editing. Reducing the occurrence of gene-edited alleles present in the germline of gene-edited progeny through mosaicism had a much larger effect on extending the time for allele fixation than gene-editing efficiencies and zygote survival rates. Therefore, the output of these models suggests that in order to optimise gene-editing programs, reducing the occurrence of mosaicism should be the primary concern [43,44]. Although a single genotype can confer resistance, given the high rate of IAV mutation and its adaptative ability, targeting only a single gene would be a high-risk strategy due to the likelihood of mutations arising that circumvent host resistance mechanisms [46].

### 4.2. Digenic Modelling

For the ANP32 gene family swIAV resistance targets in pigs, both mutant genes were recruited in the same process by swIAV for improving genome replication efficiency. Therefore, in our simulations all four recessive alleles were necessary for phenotypic resistance to swIAV infection. In an ideal scenario, the editing of two host genes encoding proteins that are exploited by discrete steps in the viral life cycle, such as a cell surface receptor (Sialic Acid for swIAV) and a protein that is recruited to assist viral genome replication (ANP32A) would create two distinct barriers to reinfection [17,21].

In our digenic modelling the efficiency of gene-editing had a greater effect on the model outputs than when only a single gene was targeted. However, as with a monogenic target, reducing mosaicism should be prioritised over improving the efficiency of gene-editing to maximise economic and genetic benefits. The chromosomal location of the target genes was observed to have only minor effects on the genetic progress of commercial pigs and the time to fixation of resistance alleles in breeding animals between linked or independent inheritance of resistance alleles. Notably, the effect of mosaicism was more pronounced for the lower efficiency gene-editing techniques.

### 4.3. Gene-Editing Techniques

For all gene-editing methods described, it is important to emphasise that illustrative parameters were used, and that these can vary widely between target sites and protocols. Data available on gene-editing in porcine zygotes is limited and highly variable, with continual optimisation being performed on what are still relatively novel techniques [47,48].

The AAV-based systems in particular are likely to require significant optimisation to be translated from rodent zygotes and porcine somatic cells to porcine zygotes in order to be feasible and practical in a commercial setting [29,49,50]. Hurdles to AAV in vivo may arise from repeated application in dams due to a potential immune response elicited after the first attempt due to the significant number of viral vectors needed in a porcine oviduct for the technique to be effective. While it may not be AAV in vivo that becomes the primary intrauterine gene-editing method in livestock, it is likely that a technique whereby CRISPR-Cas9 can be assimilated into the AI protocols would be popular due to ease of integration with current breeding techniques.

Previous gene-editing models have included Somatic Cell Nuclear Transfer (SCNT) as a method. However, the technical expertise, time and limitations in its scalability led to it not being considered a viable commercial strategy in pigs. However, there are significant benefits of SCNT, including no gene-editing-related mosaicism in progeny, which we have described as the major limiting factor to commercial gene-editing success [51]. Microinjection also requires highly trained personnel, specific micromanipulation equipment and a trained operator for gene-editing reagents to be injected into each zygote individually, making it less suitable for the scale required in commercial pig breeding.

### 4.4. Pig Breeding

The multi-nucleus pyramid structure of pig breeding makes it particularly attractive for gene-editing programs, as alleles can efficiently flow down by selection to the Finisher herd, reducing the number of genome-edited animals required. The model was designed to be adaptable to other species with pyramid breeding systems such as chickens. Without genotyping, gene-editing would not be viable at the scale necessitated by commercial pig farming. Given that the use of genomic technologies and genotyping is already standard practice in the Nucleus and Production tiers of breeding pigs [52], additional genotyping of swIAV resistance alleles could be readily incorporated with current breeding practices.

Although there was no direct measurement of inbreeding, the population structure and selection criteria applied (nested breeding) can result in lower levels of inbreeding [37]. Bastiaansen et al., 2018 observed that the continual introduction of novel alleles by gene-editing reduced the repetitive use of dams and sires when simulating gene-editing in dairy cattle. Herds with gene-editing had lower inbreeding rates compared to when only genomic selection was applied, due to the expanding pool of animals available for selection with a genotype of interest [24].

This modelling presented here was designed to be illustrative of how genetic progress, as defined by traditional indexes assessing maternal, carcass and productivity traits is impacted by prioritisation of resistance allele selection over an aggregated genetic index and how this will affect the economic outcomes of each gene-editing strategy, as opposed to being a genuine reflection of gene-editing in a specific herd. Despite being generalised and not designed around industrial information, we do not consider this to affect the relevance of the data. The modelling code is adaptable to different breeding herds for more relevant data to a particular business if more accurate advice were to be required.

For this simulation data to have more relevance to pig breeding, the commercial application of gene-editing in pigs for human consumption will need to be legislated for. Policy that allows gene-edited organisms into the food chain has already been passed in nations such as Japan, Brazil, Australia, Argentina and Canada. The legislation in these nations does not suggest that gene-edited products must be marketed differently if the genetic edit could have been introduced through natural breeding techniques. Identification of naturally occurring swIAV resistance alleles that target two distinct pathways of viral propagation, the likelihood of market approval will be improved, and the prospect of resistance emergence will be reduced compared to if a single, novel allele is introduced. Although no porcine-related products are awaiting immediate market approval, the gene-edited PRRSV-resistant pig is currently in development for introgression into a leading swine production herd.

### 4.5. Economic Perspectives

The financial outlay required to gene-edit pigs at a commercial scale will be high, particularly if the strategy involves targeting multiple genes. Our model determined the greatest costs of a gene-editing program to be not from the gene-editing procedure itself, but from unrealised gains including the loss of genetic progress compared to a herd breeding under status quo conditions and from fewer pigs reaching slaughter because of the zygote handling and gene-editing protocols resulting in smaller litters.

The economic analysis uses data from an experimental setting for the R0 value [43], fixed gene-editing costs extrapolated from application in research and a specific value for the annualised financial benefit of genetic improvement. These parameters will vary according to the farm region and system of interest. As a result, it may be quicker to reach herd immunity at a lower cost, which would affect the final decision-making process and not be directly replicated by the data presented here. However, this analysis still provides a preliminary basis for identifying the method of optimal financial efficiency when implementing a gene-editing program in commercial pigs.

The selection accuracies simulated reflect the accuracy of BV index selection in real farming systems [53]. The implications observed regarding accuracy when considering the practical implementation of a gene-editing program are that as selection accuracy increases, there will be a marginal reduction in the improvement of genetic merit compared to an un-edited herd. These marginal changes are contained within the economic analysis but do not alter the time by which the gene-editing methods reach a positive financial return.

In farm systems with vaccination programs, the cost of editing must be low and mosaicism negligible for even a monogenic target to reach a positive return on investment. For digenic targets, due to the longevity of the gene-editing programs, the benefits of high gene-editing efficiency outweighed the benefit of the low cost but lower efficiency. The slower dissemination of swIAV resistance alleles associated with low gene-editing efficiency was also observed when modelling the implementation of gene-editing in dairy cattle herds [23,24]. The results from the digenic modelling suggest that reaching fixation of the resistance alleles in breeding animals as quickly as possible and then continuing selection based upon genetic merit provides a better value proposition than persistent low efficiency editing that was observed to be associated with a prolonged reduction in genetic progress. To assess the economic situation relevant to a specific real-life situation for swIAV resistance, we would recommend running the simulation model with user-defined input data for gene-editing efficiency, zygote death and costs specific to the target sites and experimental protocols in place as well as interest rates and further economic factors relevant only to specific cases.

A benefit of swIAV-resistant pigs in a herd that was not included in our economic analysis is the fact that their presence is likely to reduce the prevalence of other infectious agents of PRDC [11,54]. This will lead to indirect reductions in veterinary costs and improvements in animal welfare standards and productivity. Another factor not included are regulatory and bureaucratic hurdles that will be faced when creating gene-edited swIAV-resistant pigs for the first time that are a likely to be a significant exclusion [55,56]. Our analysis does not encompass every factor, but the data provides an initial framework for economic considerations.

The benefits of controlling swIAV should not be considered in isolation to pig farming, due to the zoonotic implications for human health and other IAV-affected species [57,58]. Each pig that is swIAV-resistant is removed from the ecosystem as a potential “mixing vessel” and therefore reduces the likelihood of a new IAV strain emerging by genomic reassortment and becoming a pandemic strain after transmission to humans. Although it is a difficult to define due to the unpredictability of pandemic emergence and severity, it could be of great value to public health and macroeconomic performance in the instance that an event such as the 2009 swine influenza zoonoses is mitigated.

## 5. Conclusions

The results of our simulation model have highlighted the challenges of gene-editing two targets in a commercial pig breeding population. Monogenic resistance had considerably fewer negative genetic and economic impacts but will be more likely to be rendered ineffective by viral mutation. For all scenarios, higher levels of mosaicism and lower gene-editing efficiencies had a negative effect on the genetic merit value of pigs received by producers and increased the time to reach the HI threshold. The translation of gene-editing from a research environment to commercial livestock breeding could be transformative for animal welfare and production, and the opportunity to control the spread of IAV by reducing the role of pigs as a zoonotic transmission node could greatly benefit human health. These results highlight the need for protocol optimisation and further work to be done in improving gene-editing protocols for economically viable translation to livestock zygotes.

## Figures and Tables

**Table 1:** Summary of the parameters used for breeding functions in the simulation model. All age and time values are reported in 28-day batches.

Parameter	Value (In Batches)
Sow gestation length	4
Farrowing interval	5
Gilt age at first mating	8
Boar age at first mating	8
Litter size (No of piglets)	12

**Table 2:** Parameters for gene-editing functions used in simulation models. Gene-editing costs based are based on research lab data (personal communication from Dr Chris Proudfoot).

Gene-Editing Method	Editing Efficiency	Zygote Survival	Cost per Zygote	Sources
Microinjection	37.5%	40%	$100	[26]
Electroporation	60%	25%	$80	[27]
Adeno-associated Virus ex vivo	90%	15%	$80	[29]
Adeno-associated Virus in vivo	20%	75%	$10	[29]

**Table 3:** A summary of the parameters relevant to the economic analysis of the simulation results. All monetary values are quoted in US dollars.

Parameter	Value
Influenza A Virus (IAV) Productivity Loss/Pig (41)	$6.60
IAV Vaccination Cost/Pig (41)	$3.71
Annual Genetic Improvement/Pig	$4
Herd Immunity (43)	90%
Interest Rate/Annum (df)	5% (0.05)
Editing Efficiency	Variable for gene-editing method (Table 2)
Zygote Death Rate	Variable for gene-editing method (Table 2)
Cost per Zygote	Variable for gene-editing method (Table 2)
Pig Market Value (40)	$109.5

## Data Availability

Not applicable.

## Supplementary Materials

The following supporting information can be downloaded at: https://www.mdpi.com/article/10.3390/genes13081436/s1, Figure S1: Monogenic resistance with 50% germline transmission; Figure S2: Monogenic resistance with 1 and 0.5 accuracy and 100 or 20% mosaicism; Figure S3: The proportion of phenotypically resistant pigs in the Finisher herd in a gene-editing scenario of digenic swIAV resistance with 1 and 0.5 selection accuracy; Figure S4: Number of zygotes attempted to be gene-edited in the Nucleus tier in a digenic gene-editing program for linked and independent inheritance; Figure S5: Genetic merit trend of piglets in the Finisher herd in a digenic gene-editing program with linked allele inheritance; Figure S6: Economic analysis of farm systems with vaccination programs for linked swIAV resistance alleles for varied selection accuracies; Figure S7: Economic analysis of farm systems with no vaccination program for linked swIAV resistance alleles for varied selection accuracies.
