# Supplementary material for: Simulating the Commercial Implementation of Gene-Editing for Influenza A Virus Resistance in Pigs: An Economic and Genetic Analysis
Source: Genes (Basel). 2022 Aug 12;13(8):1436. doi: 10.3390/genes13081436 (PMC9407728; doi:10.3390/genes13081436)
Supplement: Supplementary file 1 [file genes-13-01436-s001.zip › Figure S1.pdf]

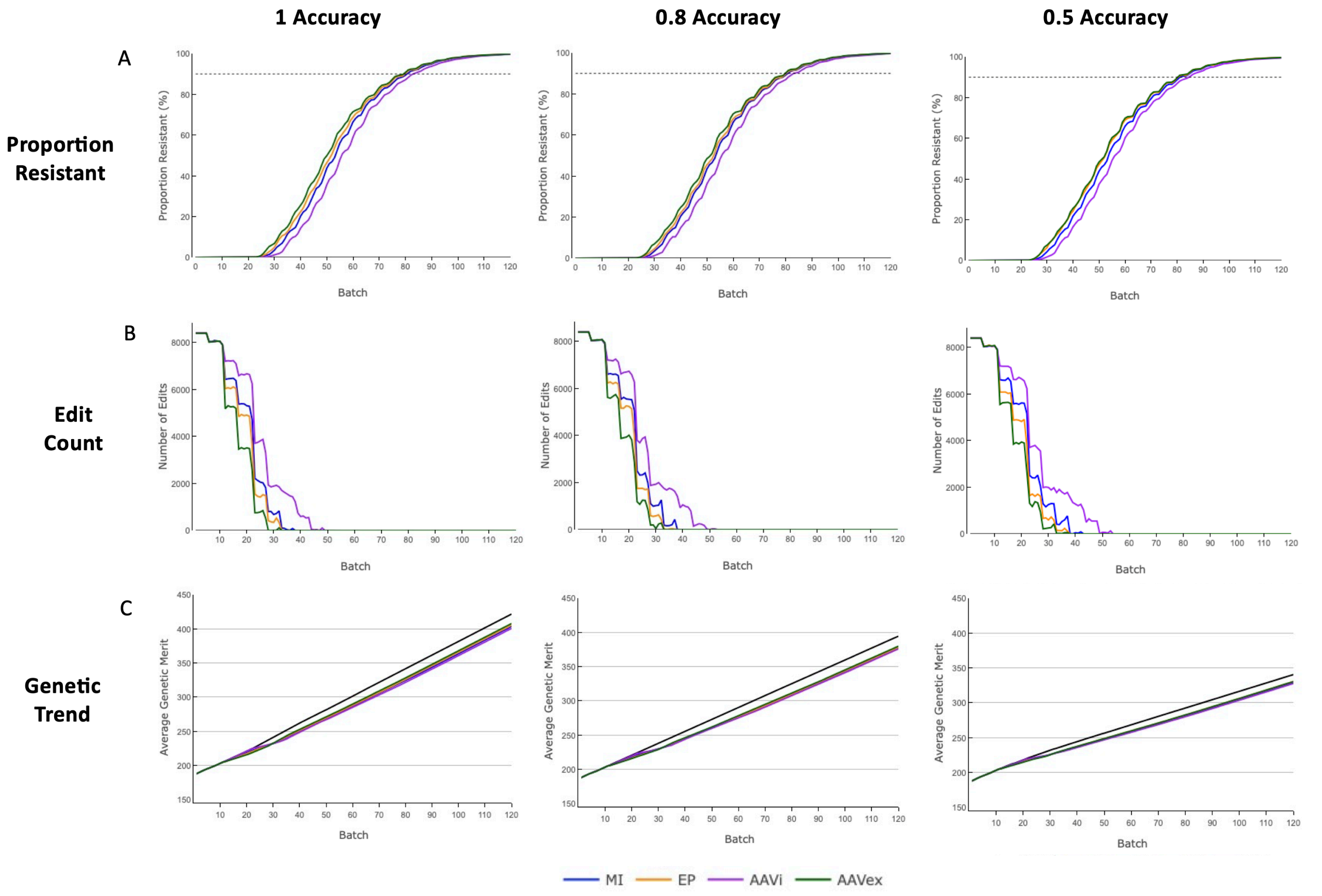

Figure S1: Monogenic resistance with 50% germline transmission. MI = Microinjection. EP = Electroporation. AAVi = AAV *in* vivo. AAVex = AAV *ex* vivo. A) Proportion of pigs with phenotypic resistance to swIAV in the Finisher Herd. B) Total count of zygotes attempted to be gene-edited across all Nucleus herds per batch. C) Mean genetic merit of pigs in the Finisher Herd.
