# Supplementary material for: Simulating the Commercial Implementation of Gene-Editing for Influenza A Virus Resistance in Pigs: An Economic and Genetic Analysis
Source: Genes (Basel). 2022 Aug 12;13(8):1436. doi: 10.3390/genes13081436 (PMC9407728; doi:10.3390/genes13081436)
Supplement: Supplementary file 1 [file genes-13-01436-s001.zip › Figure S3.pdf]

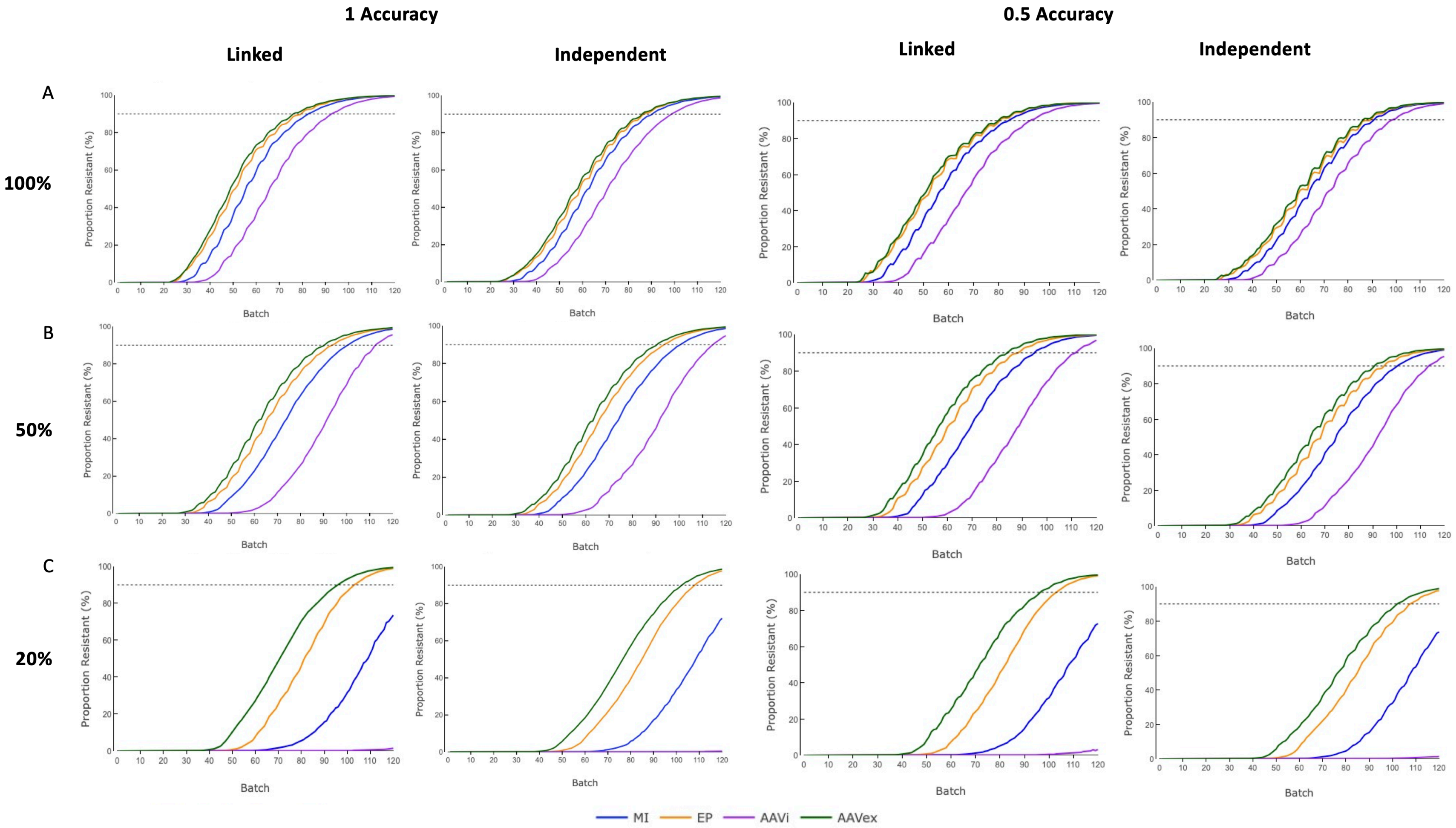


**Figure S3**: The proportion of phenotypically resistant pigs in the Finisher herd in a gene-editing scenario of digenic sIAV resistance with 1 and 0.5 selection accuracy. Changing of selection accuracy did not affect the dissemination of alleles. MI = Microinjection. EP = Electroporation. AAVi = AAV *in* vivo. AAVex = AAV *ex* vivo. Alleles were inherited in a completely linked or independent manner. A) 100% mosaicism. B) 50% mosaicism. C) 20% mosaicism.
