# Supplementary material for: Simulating the Commercial Implementation of Gene-Editing for Influenza A Virus Resistance in Pigs: An Economic and Genetic Analysis
Source: Genes (Basel). 2022 Aug 12;13(8):1436. doi: 10.3390/genes13081436 (PMC9407728; doi:10.3390/genes13081436)
Supplement: Supplementary file 1 [file genes-13-01436-s001.zip › Figure S4.pdf]

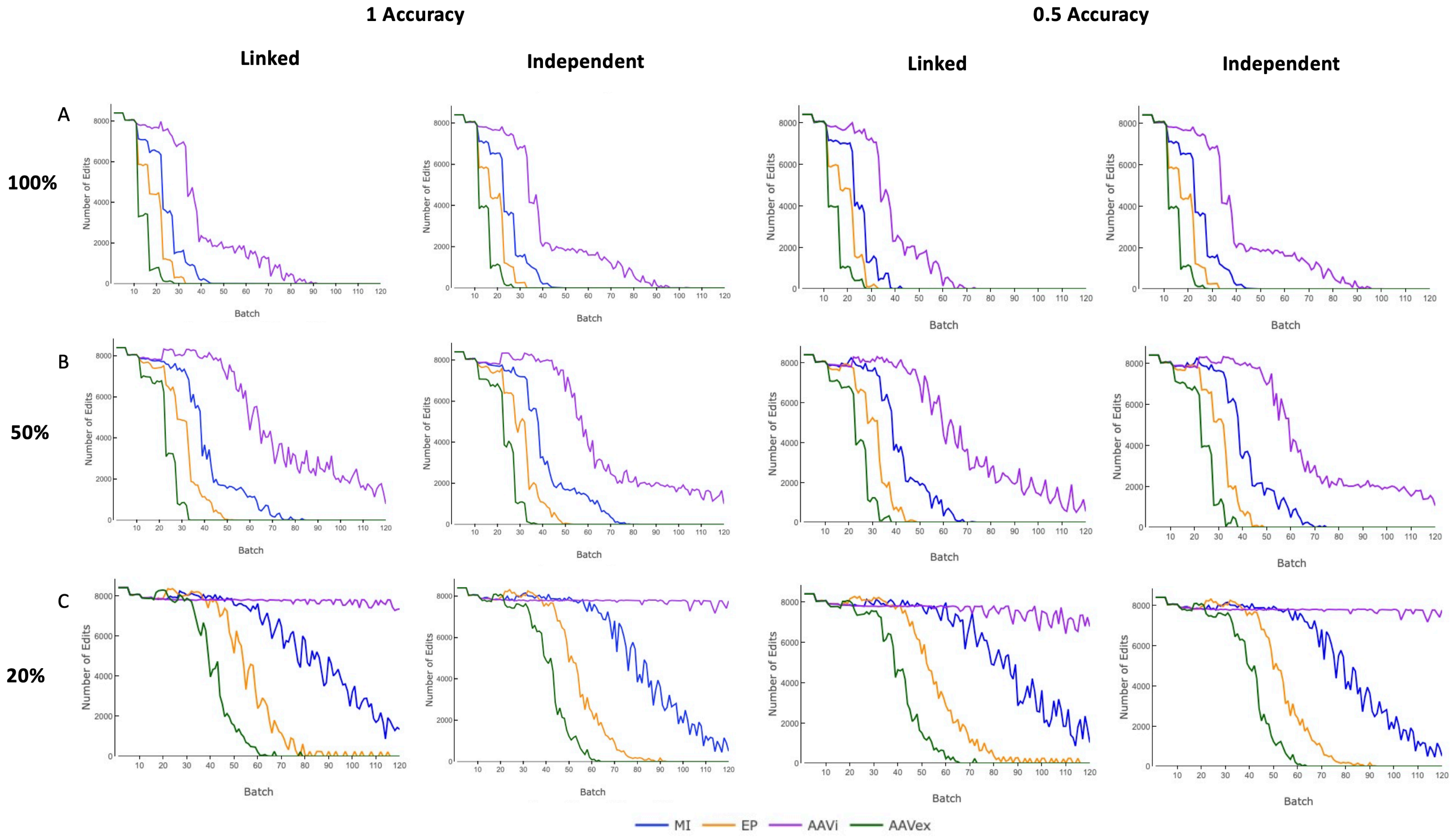

Figure S4: Number of zygotes attempted to be gene-edited in the Nucleus tier in a digenic gene-editing program for linked and independent inheritance. MI = Microinjection. EP = Electroporation. AAVi = AAV *in* vivo. AAVex = AAV *ex* vivo. A) 100% germline transmission. B) 50% germline transmission. C) 20% germline transmission.
