# Supplementary material for: Simulating the Commercial Implementation of Gene-Editing for Influenza A Virus Resistance in Pigs: An Economic and Genetic Analysis
Source: Genes (Basel). 2022 Aug 12;13(8):1436. doi: 10.3390/genes13081436 (PMC9407728; doi:10.3390/genes13081436)
Supplement: Supplementary file 1 [file genes-13-01436-s001.zip › Figure S7.pdf]

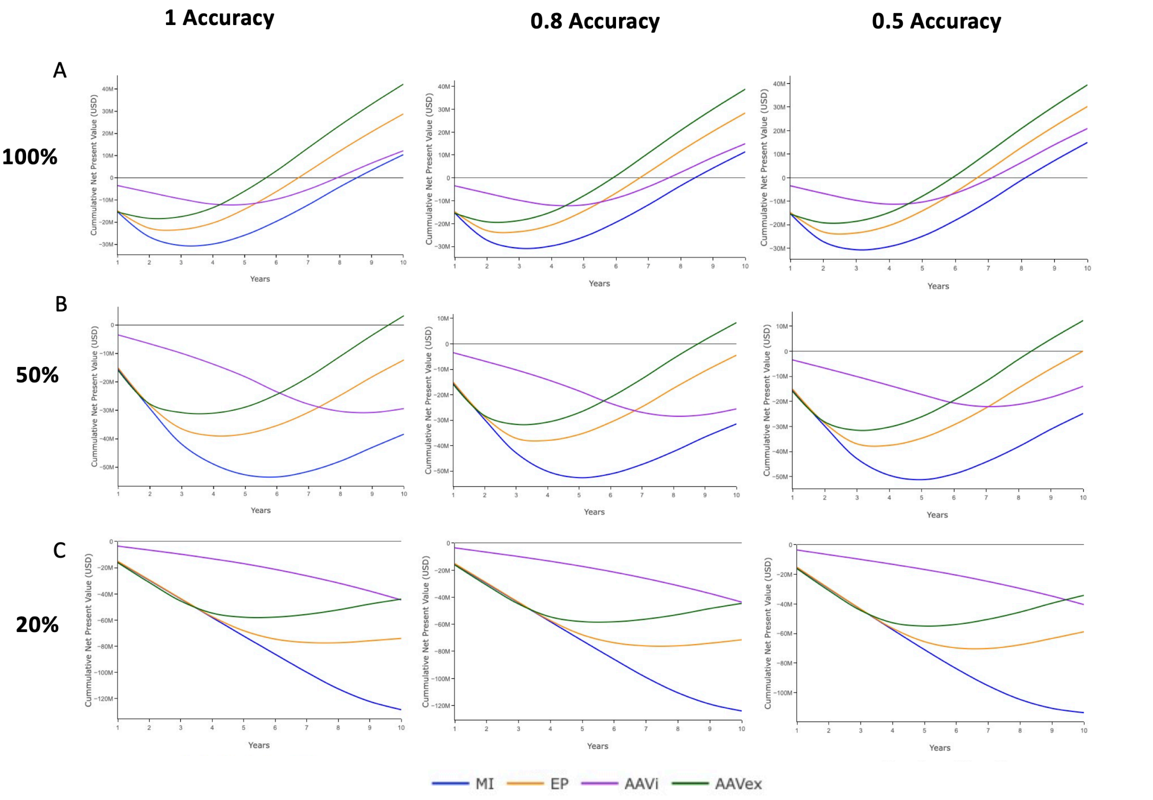

Figure S7: Economic analysis of farm systems with no vaccination program for linked swIAV resistance alleles for varied selection accuracies. MI = Microinjection. EP = Electroporation. AAVi = AAV *in* vivo. AAVex = AAV *ex* vivo. A) 100% germline transmission. B) 50% germline transmission. C) 20% germline transmission.
